# The Effect of the Degree of Astigmatism on Optical Quality in Children

**DOI:** 10.1155/2017/5786265

**Published:** 2017-06-01

**Authors:** Jing Gao, Xiao-xia Wang, Lin Wang, Yuan Sun, Rui-fen Liu, Qi Zhao

**Affiliations:** Department of Ophthalmology, The Second Affiliated Hospital of Dalian Medical University, Dalian, Liaoning Province, China

## Abstract

**Purpose:**

To investigate the effect of the degree of astigmatism on optical quality in children. The important objective evaluation parameters we focus on include the RMS of the high-order aberrations, MTF, and PSF.

**Methods:**

The children, age ranging from 7 to 10 years old, underwent an optometry examination. Fifty-nine children who met the inclusion criteria were divided into three groups: A (1.0 D ≤ astigmatism < 2.0 D), B (2.0 D ≤ astigmatism < 3.0 D), and C (3.0 D ≤ astigmatism < 4.0 D). The OPD-SCAN-III aberrometer was used to measure PSF, MTF, and other optical parameters. Total higher-order aberrations, total coma aberrations, total spherical aberrations, and total trefoil aberrations corresponding to the RMS value, the AR value of MTF, and the SR value of PSF with a 4 mm pupil diameter were assessed.

**Results:**

RMS-HO, RMS-T.Coma, RMS-T.Tre, and RMS-T.Sph in the three groups were significantly increased with increasing the degree of astigmatism, while there were no significant differences in RMS-T.Sph between the groups. The AR value and the SR value decreased with increasing degree of astigmatism, and there were significant differences in the AR value and the SR value.

**Conclusion:**

Astigmatism has a significant influence on the higher-order aberrations, MTF, and PSF in the children. The effect of astigmatism value on the optical quality is mainly reflected in the change in these three parameters.

## 1. Introduction

Eye is an important human sensory organ that receives 2/3 of the total external sensory information [1]. All people wish to have good visual acuity and visual quality. Any changes of the optical system make influence on visual quality. However, the research on optical quality is mainly focused on wavefront aberration [2, 3], few data demonstrating the relationship between wavefront aberration and the optical quality of children.

Astigmatism, one of the defects of the optical system, has a great effect on the development of visual acuity [4]. Recently, researchers have been paying more attention to the development of children's optical quality [5, 6]. Thus, we aimed to evaluate the influence of the degree of astigmatism on the optical quality in children. The evaluation parameters used in our study include the RMS of higher-order aberrations (HOAs), MTF, and PSF.

## 2. Methods

### 2.1. Research Objects and Groups

The study followed the tenets of the Declaration of Helsinki. We analyzed the clinical characteristics of children with with-the-rule astigmatism diagnosed from August 2015 to April 2016 at the Pediatrics Department of Ophthalmology Center, the Second Affiliated Hospital of Dalian Medical University. All patients met the following criteria: (1) age between 7 and 10 years; (2) with-the-rule astigmatism with cylindrical refraction more than or equal to 1.0 diopters (D) and spherical refraction less than 1.50 D; (3) best-corrected visual acuity more than or equal to 0.8; (4) transparent cornea, no corneal macula or corneal nebula, and no keratoconus; (5) corneal contact lens: soft lens removed more than a week and hard lens removed more than two weeks; and (6) no history of glaucoma; corneal, cataract, or retinal diseases; or any other medical diseases likely to affect vision. There is mirror symmetry between the left and right eyes of the same individual [5, 7], so we used the right eye as the research object to avoid the interference. In this study, there was no use of any eye drops except 1% atropine.

34 males (34 eyes) and 25 females (25 eyes) who met inclusion criteria were stratified into three groups based on the degree of their astigmatism: group A (25 eyes), with astigmatism greater than or equal to 1.0 D and less than 2.0 D; group B (19 eyes), with astigmatism greater than or equal to 2.0 D and less than 3.0 D; and group C (15 eyes), with astigmatism greater than or equal to 3.0 D and less than 4.0 D. Clinical data included patient age at initial visit, gender, best-corrected visual acuity (BCVA), alternate cover test, slit lamp and fundus examinations, and cycloplegic refraction.

### 2.2. Instruments

We used the NIDEK (AR-330A type) automatic optometry instrument to assess cycloplegic refraction. The OPD-SCAN-III aberrometer (Nidek Technologies, Japan) is the latest device of OPD-Scan system and includes fully integrated aberration functions such as keratometry, corneal topography, optometry, and pupil analysis.

Automatic optometry and OPD-SCAN-III were handled by the same skilled physician. Drops of 1% atropine were administered three times daily for 3 days. On the fourth day, cycloplegic refraction was performed, after which the measured results with high repeatability were chosen as the final value. The OPD-SCAN-III aberrometer was used to measure ocular aberrations, PSF, MTF, and other optical parameters. Next, the HOAs, PSF, and MTF were analyzed using RMS values, the SR value, and the AR value, respectively. Total HOAs, trefoil, coma, and spherical aberrations were obtained under the condition of a pupil diameter of 4.0 mm. Children's eyeballs have a large amplitude, and accommodation affects the optometry and aberration values. Therefore, these measurements should be carried out after cycloplegia. Atropine (1%) is a commonly used cycloplegic eye drop in ophthalmic work.

### 2.3. Statistical Analysis

Statistical analysis was performed using SPSS version 17 Statistical Software for Windows (SPSS Inc., Chicago, IL, USA). The results are expressed as the mean ± standard deviation. The results (RMS-HO, RMS-T.Sph, RMS-T.Coma, RMS-T.Tre, AR value, and SR value) are in accordance with a normal distribution. The differences among the three groups between the changes in the results (RMS-HO, RMS-T.Sph, RMS-T.Coma, RMS-T.Tre, AR value, and SR value) and astigmatism were compared by one-way ANOVA. A *P* value of less than 0.05 was considered statistically significant.

## 3. Results

In group A, the mean astigmatism value is 1.47 ± 0.2 D and the mean age is 8.18 ± 1.07 years old. In group B, the mean astigmatism value is 2.21. ± 0.31 D and the mean age is 8.39 ± 1.0 years old. In group C, the mean astigmatism value is 3.40 ± 0.30 D and the mean age is 8.11 ± 1.02 years old.

Higher-order aberrations changed as astigmatism changed. One-way ANOVA was performed to evaluate the relationship between astigmatism and higher-order aberrations of the three groups. The results are shown in Table 1. It was found that RMS-HO, RMS-T.Coma, and RMS-T.Tre increased as the astigmatism values increased. Further, there were significant differences between the three groups (*P* < 0.05). While RMS-T.Sph increased as the astigmatism values increased, there was no significant difference between the three groups (*P* > 0.05).

Further analysis demonstrated that there was a statistically significant difference in RMS-HO between group A and group C (*P* = 0.00). However, there was no significant difference in RMS-HO between group A and group B (*P* = 0.071) or between group C and group B (*P* = 0.058). There was a statistically significant difference in RMS-T.Coma between group A and group C (*P* = 0.00) and between group B and group C (*P* = 0.002). There was a statistically significant difference in RMS-T.Tre between group A and group C (*P* = 0.001) and between group B and group C (*P* = 0.007).

One-way ANOVA was performed to evaluate the relationship between astigmatism and the AR value of the MTF curve of the three groups. The results are shown in Table 2. It was found that the AR value increased as the astigmatism values increased. There was a significant difference between the three groups (*P* = 0.004). Furthermore, there was a statistically significant difference in the AR values between group A and group C (*P* = 0.002). However, there was no significant difference in the AR values between group A and group B (*P* = 0.099) or between group C and group B (*P* = 0.433).

One-way ANOVA was performed to evaluate the relationship between astigmatism and the SR value of the PSF curve of the three groups. The results are shown in Table 2. It was found that the SR value increased as the astigmatism values increased. There were significant differences between the three groups (*P* = 0.013). Furthermore, there was a statistically significant difference in the SR values between group A and group C (*P* = 0.001), but there were no significant differences in the SR values between group A and group B (*P* = 0.664) or between group C and group B (*P* = 0.072).

## 4. Discussion

Subjective methods for measuring visual outcomes include visual acuity (VA) and contrast sensitivity (CS). Visual acuity reveals the resolution ability of the macular to small objects with high contrast. Contrast sensitivity, which measures a person's ability to discriminate between black and white surfaces of any size, allows the human eye to identify the spatial frequency of different objects. The main problem when measuring the VA or the CS is that it requires the active cooperation of the participants, not as objective methods as wavefront aberration or objective scattering index (OSI) [8]. Wavefront aberration is a basic index for evaluating the optical quality of the human eye, and it is also the basis of other objective evaluation indexes [9], but also, MTF and PSF could be useful to evaluate objectively and quantitatively any optical system [10, 11]. The MTF curve reflects how the contrast of the object transfers to the retina [10]. The AR value is the proportion of the area defined by the curves. The ratio approximates 100%, while the curve closely approximates normal. The PSF value is determined by diffraction, aberrations, and scattering. The SR value shows the ratio of the PSF value and theoretical diffraction limitation. The ratio is close to 0.8, while it is equivalent to no aberration. As a basic tool for evaluating the imaging quality of an optical system, PSF has been widely used in the clinic, and its repeatability has been proven [12].

Wavefront aberrations can be expressed approximately by the RMS value of a Zernike polynomial [2, 13]. This study only analyzed the changes of total higher-order aberrations, total coma aberration, total spherical aberration, and total trefoil aberration corresponding to the RMS value in the children with with-the-rule astigmatism. HOAs are affected by many factors including pupil size [2, 5, 14], age [15–17], and accommodation. We used a 4.0 mm pupil to measure aberrations in children between seven and ten years old. We found that RMS-HO, RMS-T.Coma, RMS-T.Tre, and RMS-T.Sph significantly increased as with-the-rule astigmatism values increased. Hu et al. [14] found that astigmatism had an effect only on coma aberration or secondary coma aberration but no effect on secondary spherical aberration or spherical aberration in pupils with different diameters. Choi and Chang [6] found that coma aberration had a statistically significant correlation with astigmatism. These results are in accordance with our study.

Guirao et al. [18] found that corneal aberrations increased with age but did not reach the level required to reduce MTF. Zheng et al. [19] reported that aberration was the most important factor in the optical system and was closely related with MTF, such that the effect of the lower order aberration on the optical quality was much larger than that of the higher-order aberrations. This study's inclusion criterion demanded that spherical refraction be less than 1.50 D. There was no difference in spherical refraction among the three groups, so the effect on defocus in the SR value and AR value of lower order aberrations can be excluded. Thus, this study only considers the influence of astigmatism. This study finds that the AR value of the MTF curve and the SR value of the PSF curve decrease as astigmatism values increase. Furthermore, there was a statistically significant difference in the AR values and SR values between group A and group C. This fully proves that the influence of astigmatism on MTF and PSF is significant, and the variable influence is found in different degrees of astigmatism. Guo et al. [20] reported that the MTF values at all spatial frequencies decreased with increasing astigmatism, which was consistent with the results of our study. In this study, an increase relation between increasing astigmatism and higher-order aberrations, especially the asymmetry of coma aberration and trefoil aberration, was found, as well as a gradually decrease in ocular optical quality. Our results are in good agreement with previous studies relating ocular optical quality and corneal astigmatism [20–24], although there are few reports about the optical effect of increasing ocular astigmatism in children. In order to decrease the effect of high-order aberration on visual development, it is particularly important to correct the astigmatism of these children to avoid the negative effects of poor optical quality on their developing visual system.

## Figures and Tables

**Table 1:** The relationship between astigmatism and higher-order aberrations.

Parameter (mean ± SD)	Group A (*n* = 25)	Group B (*n* = 19)	Group C (*n* = 15)	*F* value	*P* value	*P*1 value	*P*2 value	*P*3 value
RMS-HO	0.148 ± 0.051	0.187 ± 0.091	0.233 ± 0.064	7.165	0.002	0.071	0.058	0
RMS-T.Coma	0.056 ± 0.024	0.062 ± 0.035	0.068 ± 0.036	8.375	0.001	0.571	0.002	0
RMS-T.Tre	0.106 ± 0.054	0.119 ± 0.064	0.176 ± 0.065	2.294	0.002	0.478	0.007	0.001
RMS-T.Sph	0.022 ± 0.019	0.03 ± 0.027	0.038 ± 0.026	6.802	0.11	—	—	—

*P*1 value indicates the comparison between group A and group B. *P*2 value indicates the comparison between group B and group C. *P*3 value indicates the comparison between group A and group C.

**Table 2:** The relationship between astigmatism and the AR and SR values.

Parameter (mean ± SD)	Group A (*n* = 25)	Group B (*n* = 19)	Group C (*n* = 15)	*F* value	*P* value	*P*1 value	*P*2 value	*P*3 value
AR value	0.217 ± 0.059	0.182 ± 0.047	0.164 ± 0.029	6.072	0.004	0.099	0.433	0.002
SR value	0.013 ± 0.009	0.01 ± 0.009	0.005 ± 0.002	4.672	0.013	0.664	0.072	0.001

*P*1 value indicates the comparison between group A and group B. *P*2 value indicates the comparison between group B and group C. *P*3 value indicates the comparison between group A and group C.
